# The relationship between visual spatial working memory capacity of tennis players and visual information processing of offensive tactical decision-making

**DOI:** 10.3389/fpsyg.2026.1562462

**Published:** 2026-01-23

**Authors:** Cheng Yufei, Wang Ningning

**Affiliations:** 1 Sport Teaching and Research Department, Zhejiang International Studies University, Hangzhou, China; 2 School of Physical Education, Northeast Electric Power University, Jilin, China; 3 Graduate Students’ Affairs Department, Shenyang Sport University, Shenyang, Liaoning, China

**Keywords:** decision-making, regions of interest, spatial working memory capacity, tennis players’ aggressive tactics, visual information processing, visual search

## Abstract

**Objective:**

To investigate how spatial working memory (SWM) ability and visual search features influence tennis players’ offensive tactical preemptive decision-making.

**Methods:**

We investigated 48 tennis players’ behavioral performance and eye movements in the decision-making offensive tactics task using a mixed experimental design of 2 (sport level: expert, novice) × 2 (SWM capacity: high-volume, low-volume).

**Results:**

(1) Tennis players with different SWM capacities had similar decision-making offensive tactics correct rates; however, expert tennis players had a significantly higher decision-making offensive tactics correct rate than the novice group, as well as a significantly lower reaction time. (2) There was variability in visual search for offensive tactical decision-making among tennis players of different skill levels. The expert group had significantly longer gaze durations, more gaze times, and greater eye-jump distances than the novice group for the three areas of interest (AOIs) of the torso, lower limbs, and racket-holding arm and racket. Conversely, the novice group exhibited fewer gaze times and shorter eye-jump distances for the two areas of interest (AOIs) of the ball and the near-court player. (3) The five areas of interest (AOI)—trunk, lower limb eye, racket arm (near-court), tennis ball, and near-court player—showed that tennis players with high SWM capacity had longer gaze durations, more gazes, and longer eye-hopping distances than the novice group.

**Conclusion:**

Spatial working memory capacity is associated with the visual search features of offensive tactical decisions, tactical decision-making agility is lower in the high-volume group, and expert group tennis players perform better in tactical decision-making and visual search strategies.

## 1 Introduction

Tennis is an open holding racket across the net confrontation project with a large field area, instantaneous transition between attack and defense, fast ball movement speed, and rich and varied technical and tactical characteristics. The above characteristics of the tennis movement make the tennis player’s ability to anticipate particularly important to its achievement of good competition (Rowe and Mckenna, 2001; Triolet et al., 2013; Hunfalvay and Murray, 2018). Expert tennis competitions are always a tug-of-war between the two players, seeking and creating winning opportunities in the transition of attack and defense over and over, so how the players can quickly search, identify, and filter key sports information, choose reasonable techniques and tactics, and master the appropriate timing of hitting the ball in a long tug-of-war is the key difficulty of tennis (Kolman et al., 2018; Bonato et al., 2019). Tennis contains a large amount of visual and spatial information, including the flight trajectory of the tennis ball, the spatial position of the two players, the direction of the opponent’s movement, the location of the court space, and some visual disturbances caused by uncontrollable factors (Furley and Memmert, 2010; Trewin et al., 2017). As a result, athletes with good anticipation ability rely on their own visual search system to recognize and track the target object in the sports situation, temporarily storing and processing the acquired visual information in the brain, and matching it with the scenario template in their own memory system, ensuring the effectiveness of processing and processing of the external visual information (Saenz-Moncaleano, 2018; Powless et al., 2020). The spatial working memory system temporarily stores and processes spatial information in the human brain. Previous studies have shown that the capacity of spatial working memory influences athletes’ decision-making and has a functional correlation with prognostic performance (Crawford et al., 2016; Giblin et al., 2016; Glavas et al., 2023).

Functional associations based on spatial working memory with athletes during anticipation have made it one of the research hotspots in the field of sport psychology in recent years (Zhang and Zhang, 2007). Previous research in sport psychology has suggested that superior decision-making efficiency is closely related to athletes’ effective processing and handling of visual information (Kassem et al., 2024). After reviewing the literature, it was discovered that the majority of current studies focus on indicators based on athletes’ attention allocation, visual processing, and training behavior, which aid in analyzing the relationship between spatial working memory capacity and athletes’ pre-conditioning performance (Jacky et al., 2015; Costa et al., 2023). For example, the study found that there would be some changes in the eye movement characteristics of athletes of different sport levels and spatial memory capacity capacities when conducting prejudgment (Matteo et al., 2017; Hinz et al., 2022). Although researchers have conducted a preliminary study on the visual search characteristics of athletes’ offensive tactical prejudgment decisions, which will shed some light on the study of explaining the functional correlation between tennis players’ attention and spatial working memory characteristics, there is still a lack of persuasive power (Renshaw et al., 2019; Klatt and Smeeton, 2022; Song et al., 2024). It was discovered that the diversity of distinct spatial working memory capacities caused diverse cognitive processing patterns in athletes’ offensive tactical decision-making, resulting in disparities in tactical decision-making performance (Harris et al., 2020; Alemanno et al., 2025). As a result, the research hypothesis was proposed that SWM capacity may be associated with the visual search features of tennis players during offensive tactical decision-making tasks. To effectively examine the mechanism of the effects of visuospatial working memory capacity on the visual information processing of athletes’ offensive tactical decision-making, the current study employed a spatial working memory task. The current study used a dual-task paradigm that included a spatial working memory task and a visual search task, as well as an expert-novice paradigm, to investigate the visual search characteristics of tennis players’ offensive tactical anticipation decisions, as well as the mechanisms by which visuospatial working memory capacity is affected in this process (Jing, 2016; Rodrigo et al., 2020; Rosker and Majcen Rosker, 2021).

To summarize, this study will begin by looking at athletes’ intrinsic mechanisms of spatial working memory capacity, using time-blocking technology and eye movement recording technology to investigate the relationship between differences in spatial working memory capacity and athletes’ anticipation performance, as well as the intrinsic mechanism of anticipation of expert athletes with varying capacities. Specifically, using the “expert-novice” paradigm, we investigate tennis players’ offensive tactical decision-making performance and visual search technique, as well as the effect of SWM capacity on offensive tactical anticipation decision-making. This research aims to help tennis players quickly master offensive tactical decision-making tactics, allowing them to improve their technical and tactical skills.

## 2 Experimental design and research methodology

### 2.1 Subjects

To verify that the sample size is reasonable, this study employed test efficacy to calculate the basic sample size for the experiment. SPSS was used to calculate the sample size. With *α* = 0.05 and a test efficacy of 0.80, the study needed a minimum of 13. The study included 48 subjects, all of whom were undergraduate and graduate students in higher education. The study divided the high and novice athletic groups based on the athletic rating with quantitative measures. In this study, 48 undergraduate and graduate students from colleges and universities were chosen, and the quantitative measure of sports level was used to divide the expert sports group and the novice sports group. There were 24 subjects in the expert group, all of whom were national level 2 tennis players (athletes who are ranked 3rd-8th in single events and 3rd-4th in teams in provincial games and championships or championships organized by the provincial sports administration department according to the Technical Ranking Standard for Tennis Players), and 24 subjects in the novice group, who had tennis experience but no athletic level. All subjects were right-handed, and they all reported understanding the “tennis offensive tactical decision making tasks” (such as net interceptions, angle attacks, and deep zone baseline attacks); they had normal visual acuity or corrected visual acuity in both eyes without astigmatism, color blindness, or color weakness; they had no symptoms of staying up all night or insomnia 1 day before the experiment; and they were in good mental health on the experiment day. Demographic and sport-specific data for all participants are summarized in Table 1. Independent-samples *t*-tests revealed that the expert and novice groups differed significantly in age, years of training, and weekly training hours (all *p* < 0.001), supporting the distinction in skill level. In contrast, no significant differences were observed between the high- and low-SWM capacity groups on these variables (all *p* > 0.05), indicating that the SWM grouping was independent of training experience.

**Table 1:** Descriptive analysis of demographic information (*M* ± SD).

Group	Number (n)	Gender (male/female)	Age (years)	Training experience (years)	Average weekly training time (hours)
Professional high-volume	12	12/0	22.08 ± 1.24	9.33 ± 1.23	17.33 ± 1.37
Professional low-volume	12	12/0	23.58 ± 1.08	10.58 ± 1.08	18.58 ± 1.08
Amateur high-volume	12	12/0	20.58 ± 1.08	4.58 ± 1.08	6.58 ± 1.08
Amateur low-volume	12	12/0	20.58 ± 1.08	4.58 ± 1.08	6.58 ± 1.08
Total	48	48/0	21.71 ± 1.58	7.27 ± 2.79	12.27 ± 5.47

### 2.2 Experimental design

A two-factor mixed experimental design with variables of motor level (expert group, novice group) and SWM capacity (high capacity group, low capacity group) was adopted. The between-group variable was motor level, while the within-group variable was SWM spatial working memory capacity. The stimulus video is broken into five Areas of Interest (AOIs): the lower limbs of the near-court player, the torso of the near-court player, the ball, the racket-holding arm and racket of the near-court player, and “our player” (referring to the overall figure of the near-court/attacking player). Tactical decision-making performance in the experimental task and oculomotor parameters of visual information processing were used as dependent variables; tactical decision-making performance included decision agility (reaction time) and decision rationality (expert evaluation), and oculomotor parameters included gaze characteristics (number of gazes, duration of gazes) and sweeping characteristics (eye-hopping distance) (Vaziri-Pashkam and Xu, 2017).

#### 2.2.1 Stimulus materials

(1) SWM capacity testing materials. To assess respondents’ SWM capacity, a recall report paradigm was utilized, as in Ahn and Lleras (2007) study. The experimental materials comprised of two types of letters, “W” and “M,” which formed a matrix of letters with n rows and m columns, and each experiment provided three “W” and n “M.” The processing load rose with the number of tests, with the pattern being that one row or column of letters was added after each experiment, and the location of “W” varied randomly and was not the same as the position in the previous experiment. The experimental material included two preliminary materials and 10 formal materials with varying processing loads.

(2) Materials for offensive tactical decision-making tasks. The “tennis offensive tactical decision making tasks” genuine video recordings were collected from 80 video stimuli of Novak Djokovic vs. Dmitry Medvedev in men’s singles with a period of 5,000 ms after screening in the pre-test, and the task system of predicting the landing place of a tennis player’s shot was self-edited by Eprime 2.0. Before the experiment, 6 tennis students and 6 minor students (non-tennis students with at least 1 semester of tennis experience) were invited to examine the validity of the stimulus material, and were asked to watch the stimulus material independently, record their decision-making, reaction time and decision rationality. Reaction time and decision rationality, edited the low-distinction video again, and repeated the test several times to finalize the stimulus material duration of 4,000 to 6,000 ms.

### 2.3 Instruments

To capture eye-movement data from each individual, a Dikablis Professional head-mounted eyetracker (Ergoneers, Germany) with a sampling rate of 60 Hz and a tracking precision of 0.1° to 0.3° was used, consistent with prior studies that have validated its application in sports-related visual search and decision-making tasks (e.g., Klatt and Smeeton, 2022; Roca André et al., 2018). The eye-camera, which tracks the eyes, and the field camera, which records the scene, were linked to the eye-tracker information collection device, and the data information transfer port was linked to a Lenovo laptop computer running D-Lab 3.0 software to record the subjects’ eye movement processes. The laptop computer with CPU model i5-11300H was linked to a SHARP XG-D3080XA projector, and the stimulus material was placed on a curtain (approximately 2.5 m from the individuals’ test area of the subjects). During the experiment, volunteers were instructed to sit and stand facing the curtain, with their eyes level with the middle of the curtain. The individuals’ eye-tracking gadget was calibrated using manual adjustments and 4-point calibration.

### 2.4 Experimental procedures

#### 2.4.1 SWM capacity testing

Subjects were brought into the laboratory in sequential order, given basic information, and then evaluated for SWM capability. Because SWM comprises of both processing and storage components, a recall report paradigm was utilized to divide people into high and poor SWM capacity. The experimental materials were two letters, “W” and “M,” arranged in a matrix of n rows and m columns, with each experiment presenting three “W” s and n “M” s.

The first test used a 3-row, 3-column matrix of WM letters, and the processing load rose with the number of tests, with the pattern of increase being the addition of one row or column of letters after each test, with the location of the “W” varied randomly and not being the same as that of the previous test. The information-processing function of the variously loaded stimulus materials was assessed using individuals’ memory judgments of the spatial placement of the letters. During each test, subjects read the information for 3,000 milliseconds before being presented with a blank screen and instructed to vocally describe the position of the three letters “W.” If the subject’s recall judgment of the letter positions was correct, the subject would proceed to the next experiment; otherwise, the stimulus material was presented again with the same load, and if the subject’s recall judgment of the letter positions was correct this time, the subject would proceed to the next test material; otherwise, the experiment was halted, and the series of the load level of the processing with the correct recall judgment would be defined as the SWM capacity. The test material consisted of 2 preparatory experimental materials and 10 formal experimental materials with different processing loads, and the length of the formal trial was approximately 3 min.

#### 2.4.2 Tactical decision-making performance tests

The preparation experiments were utilized to familiarize the subjects with the experimental protocols and operational duties, and prompt instructions were given to remedy any incorrect operations. After presenting a blank screen for 3,000 milliseconds, the stimulus material was played and subjects were asked to press keys for decision-making responses; if no key response was made, the system automatically went to the blank screen after the playback ended, and if subjects made a key response within a limited time, the system would go to the blank screen after the key was pressed. During the experiment, one person was in charge of playing back the stimulus material and recording the subjects’ vocal reports, while another was in charge of controlling the D-Lab 3.0 software. The eye movement experiment lasted around 5 min for each person.

**Figure 1:**
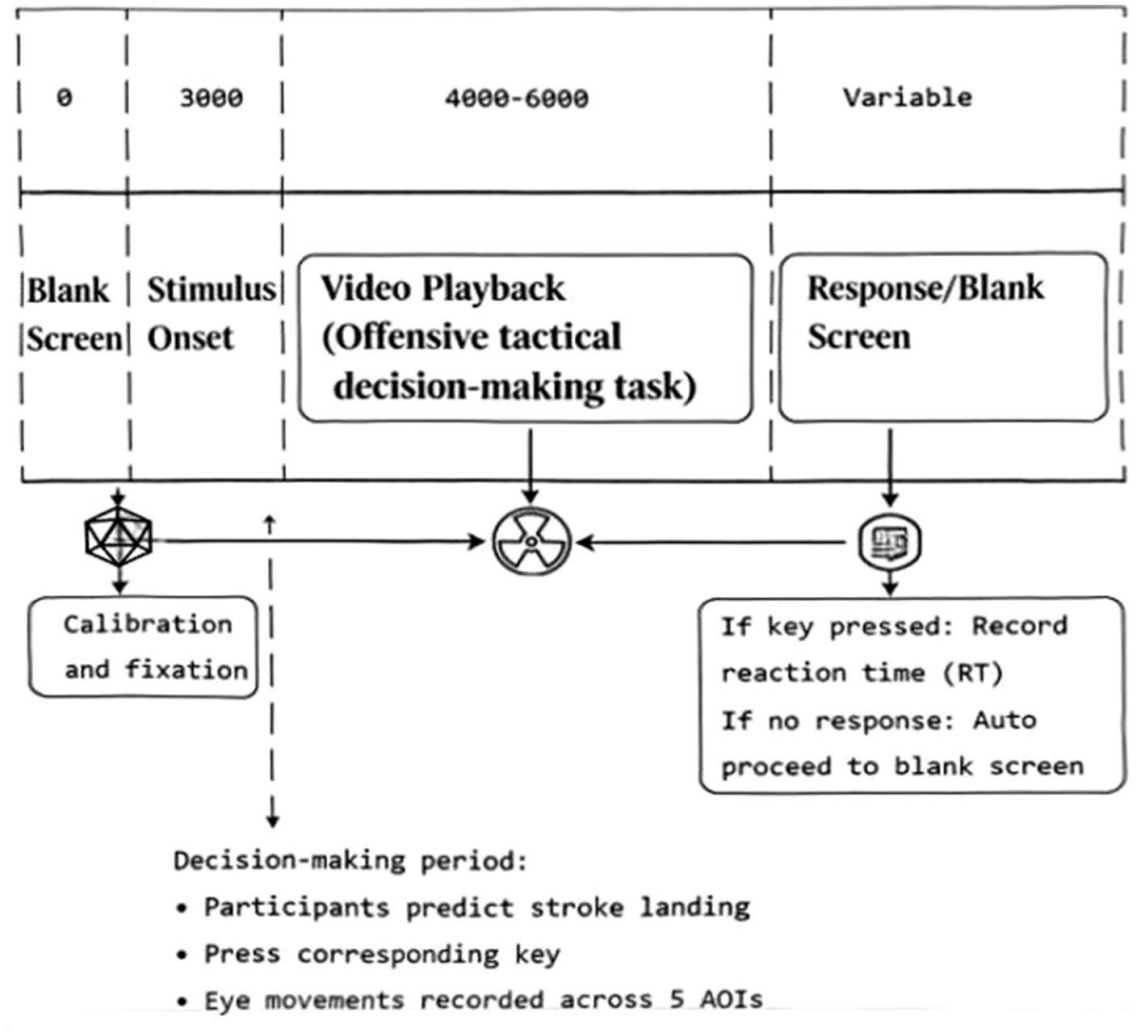
Trial procedure for the offensive tactical decision-making task.

Trial procedure for the offensive tactical decision-making task (Figure 1). Following calibration and fixation, each trial began with a 3,000 ms blank screen, succeeded by the stimulus video (4,000–6,000 ms) depicting a tennis attack scenario. During this decision-making period, participants predicted the stroke landing point by key press while their eye movements were recorded across the five predefined AOIs (lower limbs, torso, ball, racket-holding arm and racket, and our player). The trial terminated with a blank screen: reaction time was recorded if a key was pressed; otherwise, the system advanced automatically upon video completion.

(1) SWM is an important component of the multicomponent model of working memory, and it is useful for showing higher cognitive tasks like anticipatory decision-making. To effectively investigate the effect of visuospatial working memory capacity on the visual information processing of offensive tactical decision-making in tennis players, the current study draws on the work of Li and Chi (2019) and divides the subjects into high and low SWM capacity groups using the median SWM capacity as the boundary.

(2) Tactical decision-making performance is primarily defined by decision quickness and logic. In this study, tennis players’ decision-making agility is mostly indicated by the response time of their stroke landing prejudgement, whereas their decision-making rationality is primarily reflected by the right rate of their stroke landing prejudgement. Reaction time is defined as the period between the stimulus presentation and the individual’s response; the shorter the reaction time, the faster the response. As a result, reaction time is divided into two parts: the time the stimulus first appears and the time the participant responds. The current study used D-Lab 3.0 software to collect the participants’ decision response time data, which was coded according to all subjects’ tactical decision-making choices as a measure of decision-making agility. Tennis players’ decision rationality is based on the best decision-making choices in real match scenarios, and experts are invited to judge the rationality of each subject’s tactical decision-making, which is based on the source of the judgment criterion as the degree of impact that the subject’s behavioral choices have on the opposing player. The experiment used recorded video clips from professional tennis matches (Novak Djokovic vs. Dmitry Medvedev) to create stimuli for the offensive tactical decision-making task. To ensure participants could access the key visual cues used by players in real matches, the videos were presented from a third-person (side-line/broadcast) perspective, which clearly displayed the full body of the near-court (attacking) player, the ball trajectory, and the spatial relationship with the opponent. This perspective differs from a strict first-person view and was chosen to emulate the perceptual information available to a player preparing to return a shot. Based on this video perspective, five Areas of Interest (AOIs) were defined for eye-tracking analysis: (1) the torso of the near-court player, (2) the lower limbs of the near-court player, (3) the racket-holding arm and racket of the near-court player, (4) the tennis ball, and (5) the overall figure of the near-court player (referred to as “our player” in the task instructions to participants). These AOIs encompass the primary sources of anticipatory cues (e.g., body orientation, racket preparation) for predicting shot placement. To assess decision-making rationality, five experts with extensive backgrounds in tennis coaching and performance analysis were invited. They evaluated the appropriateness of each participant’s tactical choice (e.g., net interception, angle attack) for the given video scenario using a Likert 5-point scale (1 = highly unreasonable, 5 = highly reasonable). The evaluation was based on the tactical effectiveness and situational suitability of the choice within the professional match context. Finally, based on the expert evaluations awarded to the tennis players’ offensive tactical decisions, the average value was used to calculate the tactical decision reasonableness score. In addition, using D-Lab 3.0 software, the number of gaze points in each AOI was analyzed frame by frame, manually annotated, and processed to produce the eye movement characteristics needed for the study.

### 2.5 Mathematical statistics methods

Statistical analyses in this study were conducted using SPSS 25.0 (IBM Corp., United States) and Origin 21 (OriginLab Corp., United States). Primary data computation and hypothesis testing were performed with SPSS, while Origin was employed for supplementary analyses and the generation of all graphical figures. The significance level was set at *α* = 0.05. First, the consistency among the five experts in rating the rationality of tactical decision-making was assessed using Kendall’s coefficient of concordance (Kendall’s *W*). Subsequently, statistical analyses were conducted on tactical decision-making performance (reaction time, accuracy rate) and eye-movement parameters (the number of gazes, gaze duration, and saccade amplitude within each AOI). Specifically, for the multivariate eye-movement indicators, a factorial multivariate analysis of variance (MANOVA) was employed for an omnibus test. For measures that showed significant multivariate effects, as well as for the tactical decision-making performance measures, a two-way analysis of variance (ANOVA) with factors of skill level (expert/novice) and SWM capacity (high/low) was applied to examine main effects and interaction effects. All ANOVA results fully report the sum of squares, degrees of freedom, and mean square for the error term. If interaction effects were significant, simple effects analyses were performed using the EMMEANS syntax in SPSS, followed by post-hoc pairwise comparisons using the least significant difference (LSD) method, with 95% confidence intervals computed for mean differences. Additionally, partial eta-squared (η^2^) was calculated and reported as an effect size measure for all ANOVA results to evaluate the magnitude of the observed effects.

## 3 Results

### 3.1 Tactical decision-making performance

#### 3.1.1 Decision-making agility in tennis players

Tennis players’ decision-making agility is mostly indicated by their stroke landing prejudgment response time (Cardoso et al., 2020). The results of the between-subjects effect test, simple effect analysis (Tables 2–4), and pairwise comparative analysis of the tennis players’ reaction time when making a stroke landing prediction showed that the main effect of sport level was highly significant [*F*(1,42) = 307.854, *p* < 0.01, *η*^2^ = 0.880]. Furthermore, the pairwise comparative analysis of the reaction times of tennis players at different levels of athleticism when performing stroke drop precontemplation reactions revealed that the amateur group had longer average reaction times than the professional group of tennis players. Tennis players with varying spatial working memory capacities had significantly variable reaction times when executing stroke landing prejudgement [*F*(1,42) = 94.892, *p* < 0.01, *η*^2^ = 0.693]. The pairwise comparative analysis of tennis players with different spatial working memory capacities when hitting the ball to drop the point of anticipation response revealed that the difference in the mean value of the tennis players in the high-capacity group compared to the low-capacity group was between the intervals [254.183, 311.380]. Furthermore, the interaction effect between sport level and capacity group was not significant [*F*(1,42) = 0.971, *p* > 0.05, *η*^2^ = 0.0233], resulting in a non-significant simple effect of tennis players’ anticipatory response while hitting the ball on the landing place (Wright et al., 2010).

**Table 2:** Between-subjects effects test results when tennis players hit the ball on the landing spot to prejudge the response.

Source of variation	Type III sum of squares	*df*	Mean square	*F*	*p*	*η* ^2^
Sports level	2916021.304	1	2916021.304	307.854	<0.001	0.880
Volume group	898826.385	1	898826.385	94.892	<0.001	0.693
Sports level * volume group	9192.993	1	9192.993	0.971	0.330	0.023
Error	397827.288	42	9472.078			
(Grand) total	81907875.35	46				
Corrected total	3838757.747	45				

^a^*R*-squared = 0.896 (adjusted *R*-squared = 0.889).

**Table 3:** Results of the simple effects analysis during the anticipatory response of tennis players’ stroke landings.

Sports level	Volume group	Average value	Standard error	95% confidence interval^b^	
				Lower limit	Limit
Amateur	Low	1420.815	26.993	1366.341	1475.289
	High	1732.195	32.442	1666.725	1797.664
Professional	Low	940.072	29.344	880.852	999.291
	High	1194.255	26.993	1139.781	1248.729

Dependent variable: average reaction time.

**Table 4:** Results of the pairwise comparative analysis of tennis players’ responses when anticipating the landing of a stroke.

Sports level	(I) capacity group	(J) capacity group	Difference in mean values (I-J)	Standard error	*p*	95% confidence interval^b^	
						Lower limit	Limit
Amateur	Low	High	−311.380*	42.203	<0.001	−396.549	−226.212
	High	Low	311.380*	42.203	<0.001	226.212	396.549
Professional	Low	High	−254.183*	39.871	<0.001	−334.647	−173.72
	High	Low	254.183*	39.871	<0.001	173.72	334.647

**Indicates *p* < 0.01; *Indicates *p* < 0.05; ns indicates not significant (*p* > 0.05).

#### 3.1.2 Percentage of tennis players who correctly predicted the landing point of the stroke

The results of the between-subjects relate to analysis for tennis players’ accuracy in predicting ball landing points (Table 5) indicate a highly significant main effect of athletic proficiency [*F*(1,42) = 103.573, *p* < 0.001, *η*^2^ = 0.712]. This demonstrates that athletic proficiency accounts for 71.2% of the variance in accuracy, representing an extremely large effect size. The main effect of spatial working memory capacity was insignificant [*F*(1,42) = 0.013, *p* > 0.05, *η*^2^ = 0.000], indicating that the influence of capacity group on accuracy rate was negligible. The interaction between athletic level and capacity group was also insignificant [*F*(1,42) = 0.115, *p* > 0.05, *η*^2^ = 0.003] (Tables 6, 7).

**Table 5:** Results of the between-subjects effect test analysis of the correctness of tennis players’ stroke landing prediction.

Source of variation	Type III sum of squares	*df*	Mean square	*F*	*p*	*η* ^2^
Sports level	841.766	1	841.766	103.573	<0.001	0.712
Volume group	0.104	1	0.104	0.013	0.911	0.000
Sports level * volume group	0.935	1	0.935	0.115	0.736	0.003
Error	341.346	42	8.127			
(Grand) total	216903.125	46				
Corrected total	1196.603	45				

^a^*R*-squared = 0.715 (adjusted *R*-squared = 0.694).

**Table 6:** Results of the simple effects analysis of the correctness of stroke landing prediction in tennis players.

Sports level	Volume group	Average value	Standard error	95% confidence interval^b^	
				Lower limit	Limit
Amateur	Low	64.135	0.791	62.539	65.730
	High	63.750	0.950	61.832	65.668
Professional	Low	72.500	0.860	70.765	74.235
	High	72.692	0.791	71.097	74.288

Dependent variable: correctness.

**Table 7:** Results of pairwise comparative analysis of the correctness of tennis players’ anticipation of stroke landing point.

Sports level	(I) capacity group	(J) capacity group	Difference in mean values (I-J)	Standard error	*p*	95% confidence interval^b^	
						Lower limit	Limit
Amateur	Low	high	0.385	1.236	0.757	−2.110	2.879
	High	low	−0.385	1.236	0.757	−2.879	2.110
Professional	Low	high	−0.192	1.168	0.870	−2.549	2.165
	High	low	0.192	1.168	0.870	−2.165	2.549

Dependent variable: correctness based on estimation of marginal mean.

^a^Reconciliation of multiple comparisons: least significant difference method (equivalent to no adjustment).

### 3.2 AOI visual search features during the decision-making task

An independent samples t-test was used to compare the global eye movement patterns of the high- and low-capacity groups (each *n* = 24). Table 8 shows a clear pattern: the high-capacity group had considerably more fixations (*p* < 0.001) and bigger saccadic amplitudes (*p* < 0.001), with large effect sizes. This points to a more active and comprehensive visual search technique. However, the total duration of fixations did not differ substantially between the groups (*p* > 0.05), indicating that the overall time spent processing specific points of interest was comparable.

**Table 8:** Results of independent samples *t*-test.

Metric	High-capacity group (*n* = 24)	Low-capacity group (*n* = 24)	*t*-value	df	*p*-value	Cohen’s *d*	*p*
Total fixation count	11.120 ± 0.492	10.141 ± 0.748	5.021	46	<0.001	1.451	**
Total fixation duration	1364.679 ± 97.450	1314.672 ± 110.339	1.573	46	0.124	0.448	ns
Average saccade length	2.052 ± 0.191	1.882 ± 0.150	3.667	46	<0.001	1.059	**

** indicates *p* < 0.01; * indicates *p* < 0.05; ns indicates not significant (*p* > 0.05).

#### 3.2.1 Number of AOI gaze counts

**Figure 2:**
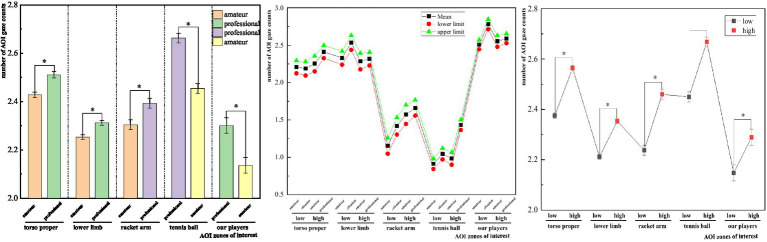
Results of a two-way multivariate analysis of variance (MANOVA) for the number of gazes during the offensive tactical decision-making task.

A multifactorial analysis was utilized to examine the between-subjects effect, simple effect, and pairwise comparative analysis of the number of area-of-interest gazes during stroke landing judgment in tennis players. The study (Table 9) found that tennis players with different memory capacities (SWM) had a significant (*p* < 0.001) effect on the number of area-of-interest gaze counts during stroke landing judgment. There was a between-subjects effect for the number of torso gaze counts (*F* = 280.128, *p* < 0.001, *η*^2^ = 0.870), a between-subjects effect for the number of lower limb gaze counts (*F* = 186.937, *p* < 0.001, *η*^2^ = 0.817), and a between-subjects effect for the number of racket gazes [*F*(1,42) = 121.772, *p* < 0.001, *η*^2^ = 0.744], a between-subjects effect for the number of ball gazes [*F*(1,42) = 193.456, *p* < 0.001, *η*^2^ = 0.822], and a between-subjects effect for the number of gazes of near-court player [*F*(1,42) = 88.944, *p* < 0.001, *η*^2^ = 0.679]. The simple effect of the number of gaze counts in the region of interest during stroke landing anticipation in tennis players with different memory capacities (SWM) showed (Figure 2) that the number of gaze counts in the high-capacity group was significantly higher than that in the low-capacity group and was more significant in the four regions of the torso gaze counts, the number of lower limb gaze counts, the number of racket-holding arm-racket gaze counts, and the number of our player gaze counts.

**Table 9:** Between-subjects effect test results for the number of eye gazes used for offensive tactical decision-making in tennis players.

Source of variation	Implicit variable	Type III sum of squares	df	Mean square	*F*	*p*	*η* ^2^
Volume group	Torso proper	6.254	1	6.254	280.128	<0.001	0.870
	Lower limb	3.817	1	3.817	186.937	<0.001	0.817
	Racket arm (near-court)	2.474	1	2.474	121.772	<0.001	0.744
	Tennis ball	3.202	1	3.202	193.456	<0.001	0.822
	Near-court player	2.421	1	2.421	88.944	<0.001	0.679
Sports level	Torso proper	1.231	1	1.231	55.151	<0.001	0.568
	Lower limb	1.880	1	1.880	92.054	<0.001	0.687
	Racket arm (near-court)	1.044	1	1.044	51.411	<0.001	0.550
	Tennis ball	0.037	1	0.037	2.236	0.142	0.051
	Near-court player	0.182	1	0.182	6.703	0.013	0.138
Sports level * volume group	Torso proper	0.348	1	0.348	15.574	<0.001	0.271
	Lower limb	0.258	1	0.258	12.620	0.001	0.231
	Racket arm (near-court)	0.094	1	0.094	4.623	0.037	0.099
	Tennis ball	0.131	1	0.131	7.924	0.007	0.159
	Near-court player	0.029	1	0.029	1.069	0.307	0.025
Error	Torso proper	0.938	42	0.022	0.938		
	Lower limb	0.858	42	0.020	0.858		
	Racket arm (near-court)	0.854	42	0.020	0.854		
	Tennis ball	0.696	42	0.017	0.696		
	Near-court player	1.143	42	0.027	1.143		

Based on estimation of marginal mean.

^a^Multiple comparison adjustment: least significant difference method (equivalent to no adjustment).

The primary influence of the number of gaze times in the region of interest of tennis players of various sport levels in the anticipation of stroke landing was more significant in the four areas of interest. The four points of interest showed significant differences in gaze times: torso [*F*(1,42) = 55.151, *p* < 0.001, *η*^2^ = 0.568], lower limbs [*F*(1,42) = 92.054, *p* < 0.001, *η*^2^ = 0.687], arm holding the racket [*F*(1,42) = 51.411, *p* < 0.05, *η*^2^ = 0.550], and player gaze [*F*(1,42) = 6.703, *p* < 0.05, *η*^2^ = 0.138]. Simple effects revealed a substantial variation in the number of times tennis players of various sports levels looked at the region of interest during the stroke landing prejudgement. For example, the number of gazes on the ball and on the near-court player during the stroke landing prejudgement was significantly higher in the expert group than in the amateur group; and the number of times tennis players of professional level looked at the torso, the number of times their lower limbs looked at the racket, the number of times their racket-holding arm and the racket looked at the racket during the stroke l were far lower than those of amateur athletes. Furthermore, the connection between sport level and SWM volume was particularly significant in four places of interest: number of torso gaze, number of lower limb gaze, number of racket arm (near-court) and racket gaze, and number of ball gaze (Wilkins and Gray, 2015; Wilkins et al., 2019; Gao et al., 2025).

#### 3.2.2 AOI gaze time

**Figure 3:**
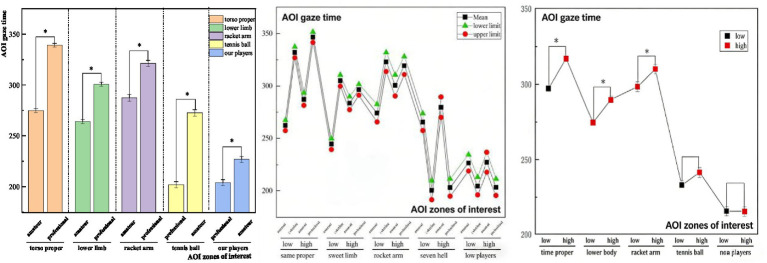
Results of a two-way multivariate analysis of variance (MANOVA) for gaze time during the offensive tactical decision-making task.

Multifactorial analyses were performed to investigate the between-subjects effects, simple effects, and pairwise comparisons of zone of interest gaze duration during stroke landing preview in tennis players. The results showed (Table 10) that there was variability in the results of the main effects of the zone of interest gaze time during stroke landing precontemplation among tennis players with different memory capacities (SWM). There was a between-subjects effect for torso proper gaze time [*F*(1,42) = 21.450, *p* < 0.001, *η*^2^ = 0.338], a between-subjects effect for lower limb gaze time [*F*(1,42) = 14.230, *p* < 0.001, *η*^2^ = 0.253], a between-subjects effect for racket arm (near-court) gaze time [*F*(1,42) = 12.670, *p* < 0.001, *η*^2^ = 0.232], whereas the between-subjects effects of ball gazing time and our player gaze time were found to be insignificant. Simple effects revealed (Figure 3) that tennis players with different memory capacities (SWM) when hitting the ball drop prediction in, torso gaze time, lower limb gaze time, and racket holding arm and racket gaze time had significant variability in the three regions, and the overall condition of the high-capacity group had significantly more gaze time than the low-capacity group (Tomohisa et al., 2004; Li and Smith, 2022).

**Table 10:** Results of the between-subjects effect test on gaze time for tennis players’ offensive tactical decision-making.

Source of variation	Implicit variable	Type III sum of squares	*df*	Mean square	*F*	*p*	*η* ^2^
Volume group	Torso proper	14285.670	1	14285.670	21.450	<0.001	0.338
	Lower limb	6952.340	1	6952.340	14.230	<0.001	0.253
	Racket arm (near-court)	5536.780	1	5536.780	12.670	0.001	0.232
	Tennis ball	4867.230	1	4867.230	3.450	0.070	0.076
	Near-court player	2789.450	1	2789.450	4.560	0.038	0.098
Sports level	Torso proper	16234.560	1	16234.560	24.370	<0.001	0.367
	Lower limb	8345.670	1	8345.670	17.080	<0.001	0.289
	Racket arm (near-court)	6234.890	1	6234.890	14.260	<0.001	0.254
	Tennis ball	1256.780	1	1256.780	0.890	0.351	0.021
	Near-court player	987.340	1	987.340	1.620	0.210	0.037
Sports level * volume group	Torso proper	2345.670	1	2345.670	3.520	0.067	0.077
	Lower limb	1567.890	1	1567.890	3.210	0.080	0.071
	Racket arm (near-court)	1234.560	1	1234.560	2.820	0.100	0.063
	Tennis ball	3456.780	1	3456.780	2.450	0.125	0.055
	Near-court player	567.890	1	567.890	0.930	0.340	0.022
Error	Torso proper	27986.450	42	666.340			
	Lower limb	20478.340	42	487.580			
	Racket arm (near-court)	18234.670	42	434.160			
	Tennis ball	59234.560	42	1410.350			
	Near-court player	24567.890	42	585.190			

Based on estimation of marginal mean.

^a^Multiple comparison adjustment: least significant difference method (equivalent to no adjustment).

Tennis players with varying athleticism levels showed significant differences in their zones of interest during stroke landing anticipation. Significant between-subjects effect tests were found for torso gaze time [*F*(1,42) = 24.370, *p* < 0.001, *η*^2^ = 0.367], lower limb gaze time [*F*(1,42) = 17.080, *p* < 0.001, *η*^2^ = 0.289], racket holding arm and racket gaze time [*F*(1,42) = 14.260, *p* < 0.001, η^2^ = 0.254]. Simple effects revealed significant variability in gaze time in the five different regions of interest during stroke landing preview among tennis players of various sport levels, with professional tennis players having significantly longer gaze times than amateurs. Furthermore, the interaction between sport level and SWM capacity was more significant at two points of interest, lower limb gaze time and racket holding arm and racket gaze time, while the other visual search interaction terms for athletes’ offensive tactical anticipation decisions were not significant.

#### 3.2.3 OI eye jump distance

**Figure 4:**
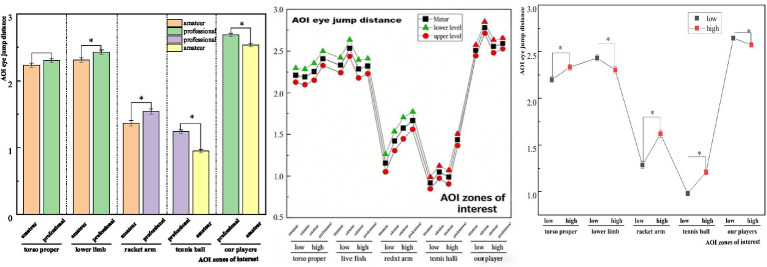
Results of a two-way multivariate analysis of variance (MANOVA) for saccade amplitude during the offensive tactical decision-making task.

Multifactorial analyses were performed to investigate the between-subjects effects, simple effects, and pairwise comparisons of zone of interest eye-jump lengths during stroke landing anticipation in tennis players. The results revealed (Table 11) that there was diversity in the outcomes of the primary impacts of the zone-of-interest eye-jump distance during stroke landing prejudgement in tennis players with varied memory capabilities (SWM). The study found a significant between-subjects effect for torso eye-jump distance [*F*(1,42) = 28.450, *p* < 0.001, *η*^2^ = 0.404], lower limb eye-jump distance [*F*(1,42) = 15.670, *p* < 0.001, *η*^2^ = 0.272], racket arm (near-court) eye-jump distance [*F*(1,42) = 45.780, *p* < 0.001, *η*^2^ = 0.522], eye-jump distance of the ball [*F*(1,42) = 52.340, *p* < 0.05, *η*^2^ = 0.555]. Simple effects showed (Figure 4) that tennis players with different memory capacity (SWM) when hitting the ball landing prediction in, torso eye-jump distance, racket-holding arm and racket eye-jump distance, ball eye-jump distance three regions with significant variability, and the overall presentation of the high-capacity group eye-jump distance is significantly higher than that of the low-capacity group; and the lower limb eye-jump distance, near-court player eye-jump distance of the high-capacity group eye-jump distance is lower than that of the low-capacity group.

**Table 11:** Results of between-subjects effect tests on eye-jump distance for offensive tactical decision-making in tennis players.

Source of variation	Implicit variable	Type III sum of squares	df	Mean square	*F*	*p*	*η* ^2^
Volume group	Torso proper	1.256	1	1.256	28.450	<0.001	0.404
	Lower limb	0.834	1	0.834	15.670	<0.001	0.272
	Racket arm (near-court)	2.345	1	2.345	45.780	<0.001	0.522
	Tennis ball	2.678	1	2.678	52.340	<0.001	0.555
	Near-court player	0.045	1	0.045	0.890	0.351	0.021
Sports level	Torso proper	0.567	1	0.567	12.840	0.001	0.234
	Lower limb	0.234	1	0.234	4.560	0.038	0.098
	Racket arm (near-court)	1.123	1	1.123	21.890	<0.001	0.343
	Tennis ball	1.456	1	1.456	28.450	<0.001	0.404
	Near-court player	0.023	1	0.023	0.450	0.506	0.011
Sports level * volume group	Torso proper	0.178	1	0.178	4.030	0.051	0.088
	Lower limb	0.067	1	0.067	1.340	0.254	0.031
	Racket arm (near-court)	0.456	1	0.456	8.900	0.005	0.175
	Tennis ball	0.789	1	0.789	15.420	<0.001	0.269
	Near-court player	0.012	1	0.012	0.240	0.628	0.006
Error	Torso proper	1.856	42	0.044			
	Lower limb	2.345	42	0.056			
	Racket arm (near-court)	2.156	42	0.051			
	Tennis ball	2.134	42	0.051			
	Near-court player	2.456	42	0.058			

Based on estimation of marginal mean.

^a^Multiple comparison adjustment: least significant difference method (equivalent to no adjustment).

Tennis players at various levels had significant differences in their zones of interest during stroke landing prediction. Torso proper eye-jump distance [*F*(1,42) = 12.840, *p* < 0.05, *η*^2^ = 0.234], lower limb eye-jump distance [*F*(1,42) = 4.560, *p* < 0.05, *η*^2^ = 0.098], racket-holding arm to racket eye-jump distance [*F*(1,42) = 21.890, *p* < 0.05, *η*^2^ = 0.343], and ball gaze time [*F*(1,42) = 28.450, *p* < 0.001, *η*^2^ = 0.404] were all significant. Simple effects revealed that there was substantial diversity in eye-jump distances in the four regions of interest of lower limbs, racket-holding arm and ball, tennis ball, and our player when tennis players of different athletic levels hit the ball to land the ball in anticipation of the ball, in which The eye-jump distances of the professional group of players were significantly higher than those of the amateur group in the two aspects of lower limbs and racket-holding arm and ball, while the eye-jump distances of the professional group of players in the two aspects of tennis ball and near-court player were significantly lower than those of the amateur group. Furthermore, the interaction between sport level and SWM capacity was more significant for the two points of interest, ball eye-hopping distance and our player eye-hopping distance, while the visual search interaction term for the remaining athletes’ offensive tactical anticipation decisions was not significant.

## 4 Discussion

### 4.1 Visual search strategies for tennis players’ offensive tactical decisions

“Fast” is a key component of the same-court rivalry program, and strong visual search is required for tennis players to respond quickly and make sound decisions. During tennis training and competition, players must quickly and accurately collect and grasp effective information related to decision-making in dynamic and changing scenes, adapt to changing external stimuli by adjusting their cognition, and make the best tactical decisions by combining their own procedural knowledge and extensive field experience (Kerstin et al., 2011; Kotegawa et al., 2024). The findings of this study indicate that SWM capacity is associated with tennis players’ offensive tactical decision-making behavior performance and visual search, supporting the claim that individual variations in SWM capacity influence decision-making behavior and immediate processing. The findings of this study demonstrated that SWM capacity significantly influenced the offensive tactical decision-making behavioral performance of tennis players (Walton et al., 2018). The tennis players in the professional group had a significantly lower stroke landing anticipation reaction time than those in the amateur group, while the players in the high SWM capacity group had a significantly higher one. This suggests that while high SWM capacity aids in information retrieval, it also requires more executive attention to resolve conflicting and interfering information, resulting in a comparatively longer information processing time; the difference in the accuracy rate of tennis players’ stroke landing prejudgment of tennis players in different SWM capacity groups and sport levels is not significant (Notarnicola et al., 2014). High SWM enables parallel processing of multiple spatial cues—such as an opponent’s body orientation and racket position—requiring athletes to screen and integrate more information rather than rely on a single heuristic cue (Mann et al., 2007). This extended pattern-matching and conflict-resolution process demands greater executive attention to suppress irrelevant cues and select optimal responses (Furley and Memmert, 2015; Rojas-Valverde et al., 2025), contributing to delayed reactions. In the current study, the expert group had higher athletic levels, longer training years, and more opportunities to participate in large and small tennis tournaments, resulting in a more complex conceptual network that included declarative and procedural knowledge related to technical and tactical skills, as well as sport-specific strategies and situational patterns (Castillo-Escamilla et al., 2025). Furthermore, the conceptual network in the expert group’s long-term memory system (e.g., specialized knowledge and number of blocks, game scenarios, and environmental changes) is an important factor in pattern recognition during motor decision-making, which can lead to comparing and matching stimulus information with existing schematic structures in the brain to effectively discriminate the meaning of that stimulus information (Guo et al., 2025). The expert group’s knowledge structure and motor schema are ordered in such a way that it can adapt its attentional strategy in response to scenario changes and swiftly seek for useful information, allowing it to make efficient and reasonable tactical judgments for tennis assault (Fleddermann and Zentgraf, 2018).

### 4.2 The association between SWM capacity and the visual search characteristics of tennis players’ offensive tactical decision-making

Tennis is a skill-based net confrontation program that combines technical, tactical, and physical abilities, necessitating great physical control and involvement from trainees. The key of success in tennis and other sports is the same: athletes must make the finest predictive decisions as fast and precisely as possible, as well as respond to the event. SWM is an essential feature of the multi-component model of working memory, which is a cognitive process that enables the temporary processing and storage of spatial information, and is important for revealing the expert cognitive activities, such as anticipatory decision-making (Klauer and Zhao, 2004). (1) The visual search for tennis players’ aggressive tactical options varied significantly across sport levels. Among them, the gaze time, gaze count, and eye-jump distance of the three areas of interest (AOI) of the expert group tennis players in the torso gaze, lower limbs gaze, and the number of times of the racket-holding arm vs. racket gaze were higher than those of the novice group tennis players, indicating that the expert group tennis players’ visual search had a more stable and in-depth focus on the body’s torso, limbs, and other aspects. As a result, inexperienced group tennis players’ visual search techniques included prolonged gaze periods for the torso, lower limbs, racket-holding arm, and ball (Jin et al., 2025). Tennis belongs to the skill-dominant group of internet confrontational sports, with the characteristics of fine technology and intense confrontation, thus tennis players must forecast and make decisions for each stroke in a limited amount of time. During the visual search process of tennis players’ offensive tactical decisions, the expert group’s attention time, number of times of attention, and eye-jump distance for the two areas of interest (AOI), namely, the ball and the near-court player, were lower than those of the novice group, indicating that the expert group tennis players had extensive combat experience (Nagano et al., 2005). According to the Neural Efficiency Hypothesis (NEH), professionals have more efficient cortical functioning during cognitive activities (Del Villar et al., 2007; Li et al., 2022). When confronted with scenarios demanding offensive tactical judgments, skilled athletes respond faster than beginners (Wang and Liu, 2018). During the visual search process of offensive tactical decision-making by tennis netball players, expert netball players require fewer cognitive resources to complete the task of recognizing the techniques of expert tennis movements, and they can quickly predict the outcome of the movements with less cognitive load, resulting in less time and number of gazes (Vaeyens et al., 2007). As a consequence, expert group tennis players demonstrated greater agility and logic in offensive tactical decision making, indicating their rapid and accurate visual information extraction and processing (Biggs et al., 2017).

(2) When SWM capacity was employed for visual search for offensive tactical decision making, tennis players with varying SWM capacities had significantly variable number of gaze times for the offensive tactical anticipatory decision-making process. Overall, tennis players with high SWM ability had longer gaze durations, gaze counts, and eye-hopping distances than novices, with the development trend seen in several areas of interest (AOI) such as the torso, lower limb eye, racket-carrying arm, tennis ball, and near-court player. These visual search differences stem from SWM-modulated cognitive strategies for information screening and tactical matching (Hidy et al., 2020; Husheer and Tanentzap, 2024). High SWM facilitates a comprehensive, multi-cue screening strategy, allowing integration of diverse spatial cues (torso, limbs, racket) for in-depth tactical pattern-matching, albeit at a higher attentional cost (Jothi et al., 2025). In contrast, low SWM leads to a heuristic, cue-selective strategy, focusing on fewer predictive cues (e.g., racket orientation) for faster but less nuanced decisions (Morey and Bieler, 2013). Individuals with strong SWM capacity are more dependent on the WM system while addressing difficulties, hence these individuals are prone to malfunction under high pressure when the pressure of a complicated job interferes with their WM system, this influences tennis players’ offensive tactical decision-making; additionally, the type of task decision-making moderates task decision-making in individuals with SWM capacity, with high-capacity individuals tending to make cognitive decisions and low-capacity individuals tending to make intuitive decisions (Babadi et al., 2021; Rusmanto et al., 2023). It can be seen that different SWM capacity affects the visual search characteristics of tennis players’ offensive tactical decision-making, which is specifically reflected in the fact that the tennis players in the high-capacity expert group adopted the strategy of “small eye jump, focused attention” and invested more attention times and time in the AOI regions of interest such as the torso, the lower limbs, the racket-carrying arm, the tennis ball, and the player in front of us, etc., while the high-capacity beginner group focused more attention and time on the AOI zones of interest such as the near-court player (Vaughan and Laborde, 2021). The high-volume beginner group focused less attention and time on the AOI zones of interest. The low-capacity expert group, constrained by the limited capacity of SWM, screened key information regions through the selective attention system based on large-scale eye hopping, and thus it was hypothesized that it could inhibit attention to peripheral changing stimuli, avoid interference and conflict among multiple information, and maintain gaze tracking on key regions (Roca André et al., 2018; Dewanti et al., 2023; Ploetzner et al., 2023). The number of gazes and duration of attention directed toward the near-court player AOI were among the most significant differences between the high-capacity and low-capacity beginner groups. The novice group had limited motor experience and lacked a comprehensive motor schema matching (AOI) of ball and near-court player gaze were in the tactical decision-making situations in their long-term memory system, making it difficult to attend to this essential information.

(3) Through cross-sectional design, this study clarified that there was a significant correlation between exercise level, SWM capacity, visual search characteristics and tactical decision-making performance. However, it must be pointed out that these findings are essentially related, and their intrinsic causal direction is still uncertain (Frappier, 2018). Two causal pathways are plausible. First, visual search may mediate the effects of SWM and expertise on decisions, whereby enhanced SWM or training promotes more efficient gaze strategies, leading to better performance (Ryu et al., 2013; González-Eirín et al., 2024). Second, SWM may directly facilitate information integration and response selection, even when visual search is controlled (Furley and Memmert, 2015; Ivosevic and Stckel, 2025). Thus, visual search could be a proximal cause of decision outcomes, while SWM likely operates both indirectly (via gaze modulation) and directly (via executive control). Future work should test these models through experimental manipulation (e.g., SWM load, gaze training) or longitudinal designs to clarify causal sequences in perceptual-cognitive expertise (Williams et al., 2011,^2020^; Mathiyarasan and Krishnamoorthi, 2024).

## 5 Research limitations

Although this study sheds light on how visuospatial working memory capacity influences visual information processing for offensive tactical decision making in tennis players, it should be noted that there are several limitations. First, the sample consisted solely of college and university students, and the performance habits of tennis players of different genders were not segmented, limiting the findings’ applicability to tennis player practice and progress. Given tennis players’ offensive tactical decision-making performance and visual search strategies, it is possible to compare the gender differences that distinguish athletes with varying levels of athleticism and spatial memory capacity; furthermore, future research should focus on cross-gender dynamics to determine whether the observed findings and relate to mechanisms are generally valid. Second, all participants were undergraduate and graduate students attending colleges and universities in mainland China. Students’ educational backgrounds, cultural literacy, and social values varied significantly across regions, and the sample size in this study was relatively homogeneous, limiting the findings’ broad applicability. Third, the experimental individuals in the study were tennis players. Because different sports have diverse techniques, tactics, and confrontation styles, offensive tactical decision-making performance and visual search strategies vary among sports. Future research should look into the visual search characteristics of athletes’ offensive tactical anticipation judgments in various sports to gain a more comprehensive understanding of these connections. Furthermore, the study did not take into account variables such as socioeconomic background, prior learning experiences, or personality traits, all of which have the potential to relate to visual search characteristics and information processing in tennis players’ offensive tactical anticipation decisions. Future studies could combine many elements to find more complicated and personalized impacts. Finally, this study followed a short-term experimental design. Longitudinal studies will help determine whether tennis players’ offensive tactical decision-making performance and visual search strategies persist over time or decline with prolonged exposure, as well as provide practical insights into tennis players’ rapid mastery of offensive tactical anticipation strategies.

## 6 Conclusion

The study’s findings demonstrated that (1) tennis players’ decision-making performance of offensive tactics is significantly impacted by varying sport levels, but SWM capacity has no discernible effect. In other words, the expert group of tennis players has a much higher correct rate of offensive tactic decision-making than the novice group, and their reaction time is significantly faster than the novice group’s; the difference between the correct rate of offensive tactic decision-making among tennis players with varying SWM capacities is not statistically significant. This suggests that high-level tennis players have quick reflexes and a high rate of accurate sports decision-making. (2) Different sport levels were used to differentiate the visual search of tennis players’ offensive tactic decision-making. The expert group’s gaze time, gaze times, and eye-hopping distances in the three areas of interest (AOI) of torso gaze, lower limb gaze, and the number of times the racket-holding arm gazed at the racket were higher than those of the novice group, while the two areas of interest (AOI) of ball gaze and our player’s gaze were in the opposite state. (3) The number of gazes used in the offensive tactical anticipatory decision-making process varies significantly across tennis players with varying SWM capacities. The development trend was observed in various areas of interest (AOI) such as the torso, the lower limb eye, the racket-carrying arm, the tennis ball, and the near-court player. In general, the tennis players with high SWM capacity had higher gaze time, gaze counts, and eye-hopping distance than the novice group. It is evident from this that tennis players’ visual search tactics in offensive tactical decision-making tasks may be related to varying SWM capacities.

## Data Availability

The original contributions presented in the study are included in the article/Supplementary material, further inquiries can be directed to the corresponding author.
