# Supplementary material for: The relationship between visual spatial working memory capacity of tennis players and visual information processing of offensive tactical decision-making
Source: Front Psychol. 2026 Jan 23;17:1562462. doi: 10.3389/fpsyg.2026.1562462 (PMC12875949; doi:10.3389/fpsyg.2026.1562462)
Supplement: Supplementary file 1 [file Table_1.docx]

**知情同意书**

尊敬的 先生/女士：

我们想邀请您参加一项《视觉空间工作记忆容量对网球运动员进攻战术决策视觉信息加工的影响》（An Examination of the Impact of Internet Word of Mouth on the Adoption Behavior of Sports and Leisure Tourism in the Digital Media Era），本研究已通过浙江外国语学院（Zhejiang International Studies University）伦理委员会审核与批准。本知情同意书将向您介绍该研究的目的、步骤、获益、风险、不便以及您的权益等，请仔细阅读后慎重做出是否参加研究的决定。当研究人员向您说明和讨论知情同意书时，您可以随时提问并让他/她向您解释您不明白的地方。您可以与家人、朋友以及您的主治医师讨论之后再做决定。

知情同意声明：

我已被告知此项研究的目的、背景、过程、风险及获益等情况。我有足够的时间和机会进行提问，问题的答复我很满意。

我也被告知，当我有问题、想反映困难、顾虑、对研究的建议，或想进一步获得信息，或为研究提供帮助时，应当与谁联系。

我已经阅读这份知情同意书，并且同意参加本研究。

我知道我可以选择不参加此项研究，或在研究期间的任何时候无需任何理由退出本研究。

我已知道如果我的状况更差了，或者我出现严重的不良事件，或者我的研究医生觉得继续参加研究不符合我的最佳利益，他/她会决定让我退出研究。无需征得我的同意，资助方或者监管机构也可能在研究期间终止研究。如果发生该情况，医生将及时通知我，研究医生也会与我讨论我的其他选择。

我将得到这份知情同意书的副本，上面包含我和研究者的签名。

受试者姓名：________________________

受试者签名：________________________

她是自愿同意的。

研究者姓名： ________________________

研究者签名： ________________________

日期：____________年____________月____________日

注：如果受试者不识字时尚需见证人签名，如果受试者无行为能力时则需代理人签
